# Perfectionism and self-worth among college student basketball referees: the chain mediating roles of self-efficacy and social anxiety

**DOI:** 10.3389/fpsyg.2026.1875231

**Published:** 2026-08-28

**Authors:** Linfeng Zou, Qiang Guo, Tong Liu, Zhonggen Yin, Mingda Li

**Affiliations:** 1 School of Sports Training, Chengdu Sport University, Chengdu, China; 2 Chongqing University of Education, Chongqing, China

**Keywords:** basketball referees, perfectionism, self-efficacy, self-worth, social-anxiety

## Abstract

**Background:**

Basketball referees work under rapid, physically demanding, and publicly evaluated conditions that may intensify perfectionistic concerns, including zero-error expectations, sensitivity to external evaluation, and recurrent self-doubt. Evidence remains limited on how perfectionism is statistically associated with self-worth in college student basketball referees and whether self-efficacy and social anxiety account for this association in sequence.

**Methods:**

A cross-sectional questionnaire survey was completed by 860 college student basketball referees aged 18–22 years from Sichuan, Chongqing, Guizhou, and Yunnan; 66.0% were male and all had at least 1 year of refereeing experience. Participants completed measures of perfectionism, self-worth, self-efficacy, and social anxiety, and the reported total scores were used in the primary analyses. Serial mediation associations were examined with SPSS PROCESS Model 6 and 5,000 bootstrap samples.

**Results:**

Perfectionism correlated negatively with self-worth (*r* = −0.354) and self-efficacy (*r* = −0.340), and positively with social anxiety (*r* = 0.342); self-efficacy correlated positively with self-worth (*r* = 0.428) and negatively with social anxiety (*r* = −0.405), whereas social anxiety correlated negatively with self-worth (*r* = −0.523). The total effect of perfectionism on self-worth was −0.2517 [95% CI (−0.2984, −0.2050)], and the direct effect remained significant after the mediators were included [−0.1313, 95% CI (−0.1781, −0.0845)]. All three indirect associations were significant because their bootstrap confidence intervals excluded zero: *via* self-efficacy, *via* social anxiety, and *via* the serial self-efficacy-to-social-anxiety pathway.

**Conclusions:**

Higher perfectionism was associated with lower self-worth among college student basketball referees, and self-efficacy and social anxiety showed significant independent and sequential indirect associations. Because the direct effect remained significant, the findings support partial rather than full mediation. The results identify potentially useful training targets, but longitudinal or experimental studies are required before causal or intervention effects can be established.

## 1 Introduction

Basketball officiating involves rapid transitions, physical contact, complex information, and immediate public feedback (Shi, 2024). Student referees must enforce rules while managing academic demands, qualification development, competition practice, and evaluation from players, coaches, spectators, instructors, and peers (Cunningham et al., 2022, 2025). Understanding their psychological adaptation is therefore relevant to referee training, wellbeing, and continued professional development (Karaçam et al., 2023). Accuracy, fairness, and composure are essential in refereeing, but these standards may become maladaptive when they are accompanied by excessive self-criticism, catastrophic interpretations of mistakes, and persistent concern about evaluation (Rajapakse, 2024). Frost's multidimensional model includes concern over mistakes, personal standards, parental expectations, organization, and doubts about actions (Burgess et al., 2016). In referees, concern over mistakes and doubts about actions may be expressed as post-game rumination, fear of negative evaluation, and reduced decisiveness under pressure. Self-worth is an individual's broad evaluation of personal value, ability, and positive qualities (Birni and Eryilmaz, 2024). For student referees, it may be shaped by academic experiences, role recognition, rule competence, on-court control, and respect from others (Crocker et al., 2006). Because officiating is public and evaluative, criticism may be internalized as a global judgment of personal worth, particularly when self-worth is unstable (Davey et al., 2003).

Self-efficacy refers to beliefs about the capacity to organize and perform actions required to achieve a goal (Stajkovic and Luthans, 1998). In refereeing, it includes confidence in rule application, problem solving, communication, game control, and adaptation to changing situations (Karacam and Adiguzel, 2019). Perfectionistic concerns may be associated with lower self-efficacy when errors are treated as evidence of incompetence and successful experiences are discounted.

Social anxiety involves tension, worry, and avoidance in situations in which a person expects scrutiny or evaluation (Zeidner and Matthews, 2005). Basketball officiating is strongly social-evaluative because decisions are public and reactions from coaches, players, spectators, and senior officials can be immediate and vocal (Adé et al., 2018; Snyder and Purdy, 1987). Perfectionism is consistently associated with social anxiety when external evaluation becomes closely tied to self-worth (Yap et al., 2016). Considering self-efficacy and social anxiety together may clarify how perfectionism is associated with self-worth. Perfectionistic concerns may coincide with weaker beliefs about officiating competence; lower competence beliefs may heighten perceived threat in public evaluation; and sustained evaluative anxiety may be associated with lower self-worth (Gaudiano and Herbert, 2007). The present study therefore tested self-efficacy and social anxiety as parallel and sequential statistical mediators.

Sport-psychology research has devoted substantially more attention to athletes than to officials, although referees' psychological states are relevant to attention, judgment, communication, stress recovery, and the quality of competition (Samuel, 2020; Zhang et al., 2022). Student referees are a particularly relevant group because they are simultaneously learners and rule enforcers and must manage both educational and in-game evaluation (Wang et al., 2022). Studying their perfectionism, self-efficacy, social anxiety, and self-worth extends referee preparation beyond technical instruction to psychological development.

Self-worth is embedded in professional roles, social interactions, and developmental contexts rather than being solely an internal feeling (Karanikola et al., 2018). Early-career student referees may rely heavily on external recognition when interpreting controversial calls, conflict, and peer comparison. High self-efficacy and a learning-oriented interpretation of evaluation may help preserve self-worth, whereas treating mistakes as unacceptable may make self-worth more contingent and fragile.

The study asks not only whether perfectionism is associated with self-worth but also whether the association is statistically accounted for by self-efficacy and social anxiety. The proposed order is theoretically consistent with a progression from evaluative standards to competence beliefs, evaluative emotion, and self-worth (Robazza et al., 2023). However, because the data are cross-sectional, the model tests an ordered statistical association and cannot establish temporal sequence or causal mechanism.

Student basketball referees are important to campus and grassroots competitions and form part of the future referee workforce in school, community, and competitive sport (Vealey and Chase, 2016). Their early assignments often require rule explanation, conflict management, and order maintenance under uneven playing conditions and limited support (Isidori et al., 2012). Because correct decisions are often unnoticed while controversial calls are highly visible, this asymmetric feedback may intensify attention to mistakes and destabilize self-evaluation among perfectionistic novices (Kaissidis-Rodafinos et al., 1997; Schrödter, 2024).

Ages 18–22 constitute an important period for the development of vocational interests, professional identity, self-concept, and social relationships (Tanner and Arnett, 2016). During this stage, high standards may support learning and professionalism, whereas excessive concern over mistakes may cause normal developmental errors to be interpreted as evidence of incompetence (Bieling et al., 2004; Osenk et al., 2020). This distinction is central to understanding why the global perfectionism score used in this study should be interpreted cautiously.

Self-efficacy and social anxiety were selected because they map directly onto common challenges in referee development: applying rules confidently and functioning under observation and evaluation (Rudy et al., 2012; Siedentop et al., 2019). Together, they link a relatively stable evaluative tendency to a higher-order judgment of self-worth and provide potentially modifiable targets for training. The study therefore evaluates the following hypotheses while retaining an associational interpretation.

## 2 Literature review and research hypotheses

### 2.1 Perfectionism and self-worth

Perfectionism involves both high performance standards and the conditions under which people grant themselves acceptance or worth (Chinoy, 2017). When self-worth depends on meeting inflexible standards, success may prompt further escalation of those standards, whereas mistakes may be interpreted as global self-failure (Crocker et al., 2006). Research distinguishes perfectionistic concerns, which are generally associated with lower self-esteem, from perfectionistic strivings, whose associations depend on the presence of self-criticism and fear of failure (Fearn et al., 2022).

High standards have adaptive value in basketball officiating, but student referees are still developing competence and professional identity. When each decision is treated as a test of personal worth, concern over mistakes may amplify the threat of live evaluation, peer comparison, and instructor feedback (Smith, 2013; Su and Chano, 2024). Accordingly, higher global perfectionism was expected to be associated with lower self-worth in this sample.

The association may be especially salient in refereeing because performance feedback is immediate, public, and sometimes ambiguous. Unlike examination scores, the same officiating decision may be evaluated differently according to viewing angle, rule interpretation, and stakeholder interest (Boyer et al., 2020). Seeking absolute certainty in such conditions may increase evaluative pressure and make self-worth more dependent on external reactions (Thompson, 1994).

Perfectionists may also engage in repeated counterfactual thinking after difficult events (Sirois et al., 2010). Reflection can support learning, but persistent rumination about a controversial call may shift from technical review to global self-condemnation. The proposed association may therefore extend beyond the game into post-game review and everyday self-evaluation (Keegan et al., 2010).

*H1:* Perfectionism is negatively associated with self-worth among college student basketball referees.

### 2.2 The mediating role of self-efficacy

Self-efficacy concerns expectations about one's capacity to act effectively and is associated with goal choice, effort, persistence, and emotion (Schunk, 2012). For referees, it encompasses confidence in recognizing key actions, applying rules, managing disputes, communicating decisions, and adapting attention as play changes (Maddux and Lewis, 1995). Higher self-efficacy may support a correctable interpretation of errors, whereas lower self-efficacy may encourage global ability attributions.

Perfectionistic concerns may direct attention toward failure cues, promote repeated checking, and make competent performance feel insufficient (Ciarocco et al., 2010). These processes may weaken the accumulation of mastery experiences that normally support referees' sense that they can handle demanding situations (MacMahon et al., 2007).

Self-efficacy is also positively associated with self-worth because competence beliefs support perceptions of developmental potential and social value (Nicholls et al., 1989). For student referees, officiating self-efficacy concerns both technical confidence and perceived value within the event and professional-development context (Lirgg et al., 2016). Self-efficacy was therefore expected to statistically mediate part of the association between perfectionism and self-worth.

Referee self-efficacy can be considered across cognitive rule judgment, behavioral execution, and social communication (Karacam and Adiguzel, 2019; Stajkovic and Luthans, 1998). These domains involve confidence in identifying fouls and violations, communicating decisions through whistles and signals, and responding professionally to questions from players and coaches.

Concern over mistakes may weaken judgment confidence, doubts about actions may undermine decisive execution, and sensitivity to external expectations may make communication more rigid or avoidant (Antony and Swinson, 2009). Thus, perfectionism may be associated not only with lower general confidence but also with lower perceived competence in specific officiating tasks (Robazza et al., 2023).

Social cognitive theory positions self-efficacy as a central cognitive-motivational-affective mechanism (Schunk, 2012). Repeated experiences of judging, explaining, and managing conflict may strengthen identification with the referee role and help individuals interpret mistakes as improvable skill issues rather than personal defects (Bandura, 1982). Self-efficacy may therefore buffer the statistical association between perfectionism and lower self-worth.

*H2:* Self-efficacy statistically mediates the association between perfectionism and self-worth among college student basketball referees.

### 2.3 The mediating role of social anxiety

Social anxiety centers on fear of negative evaluation, which is especially relevant when referees' decisions, signals, communication, and consistency are visible to others (MacMahon and Plessner, 2007). Student referees may be particularly prone to self-focused attention because of their limited experience (Neil et al., 2013). Perfectionism is associated with social anxiety when failing to meet perceived standards is interpreted as evidence of low personal worth (Wang et al., 2022).

Higher social anxiety is generally associated with lower self-worth. Anxiety increases attention to criticism and may reduce participation in challenging interactions, limiting opportunities for positive feedback and competence development (Gilbert, 1990; Schultz and Heimberg, 2008). For referees, repeated fear of criticism or rejection may gradually become a negative conclusion about personal value.

Among referees, social anxiety may appear as heightened monitoring of expressions and crowd noise, fear of communicating with coaches, avoidance of post-game discussion, or anticipatory anxiety before the next game (Vargas et al., 2025). Even when tasks are completed, persistent tension and self-monitoring can consume attention needed for the game itself.

Perfectionists may assign unusually high personal meaning to disagreement or questioning because external evaluation is treated as evidence of self-worth (McArdle and Duda, 2008). In a highly expressive basketball environment, ordinary disagreement may therefore be experienced as a threat to professional identity rather than as task-specific feedback (Liu et al., 2021).

Self-presentation and sociometer perspectives suggest that social anxiety can undermine self-worth by increasing avoidance and reducing access to positive social feedback (Maddux et al., 1988). Student referees with greater anxiety may avoid demanding assignments or detailed debriefing, which may provide short-term relief but restrict longer-term opportunities for success and growth (Gregory et al., 2016). Social anxiety may therefore serve as both an emotional correlate of perfectionism and a statistical mediator of lower self-worth.

*H3:* Social anxiety statistically mediates the association between perfectionism and self-worth among college student basketball referees.

### 2.4 Chain mediation of self-efficacy and social anxiety

Self-efficacy is theoretically related to social anxiety because stronger competence beliefs are associated with less threatening appraisals of challenging situations, whereas lower self-efficacy is associated with expectations of limited control (Bandura, 1982; Leary and Atherton, 1986). Student referees who doubt their ability to explain decisions, control play, or manage conflict may therefore anticipate more negative evaluation during games.

The proposed serial model links higher perfectionism with lower self-efficacy, lower self-efficacy with higher social anxiety, and higher social anxiety with lower self-worth (Zhang et al., 2022). This ordered model is theoretically meaningful for training, because competence-building may be more useful than simply instructing students not to feel anxious. The sequence remains a statistical ordering rather than demonstrated temporal causation.

The proposed ordering reflects a functional progression from evaluative tendency to task belief, threat appraisal, and self-worth. Theoretically, perfectionism may precede appraisals of competence, and competence beliefs may be associated with social-evaluative emotion (Liao et al., 2023; Xie et al., 2025). Nevertheless, cross-sectional mediation cannot confirm that this sequence occurred over time or exclude alternative and reciprocal pathways.

Social cognitive theory suggests that lower self-efficacy is associated with anxiety, avoidance, and sensitivity to negative evaluation, all of which may coincide with lower self-worth (Bandura, 2001). A perfectionistic student referee may enter a game fearing any error, hesitate during a contested decision, attend closely to reactions from others, and later interpret the episode as evidence of unsuitability rather than as a correctable technical problem (Campo et al., 2012). This scenario illustrates the hypothesized serial association without establishing that the sequence occurred in the present cross-sectional data.

*H4:* Self-efficacy and social anxiety form a serial statistical mediation pathway between perfectionism and self-worth among college student basketball referees.

### 2.5 Theoretical model of the present study

The theoretical model begins with perfectionism, ends with self-worth, and includes self-efficacy and social anxiety as sequential mediators. It integrates three linked propositions: perfectionistic concerns are associated with stronger self-criticism; stronger self-criticism is associated with weaker competence beliefs; and weaker competence beliefs are associated with greater anxiety under observation and evaluation (Sheikh et al., 2025). Greater social-evaluative anxiety is, in turn, expected to be associated with lower self-worth.

This model is relevant to refereeing because critical decisions are immediate, public, and difficult to verify repeatedly (Senger, 2026). A strong need to be correct may be accompanied by doubts about coping, and immediate reactions from spectators, coaches, and players may intensify perceived evaluative threat (Flett and Hewitt, 2014). Controversial decisions may then be interpreted as evidence about personal worth rather than as normal features of officiating development.

**Figure 1:**
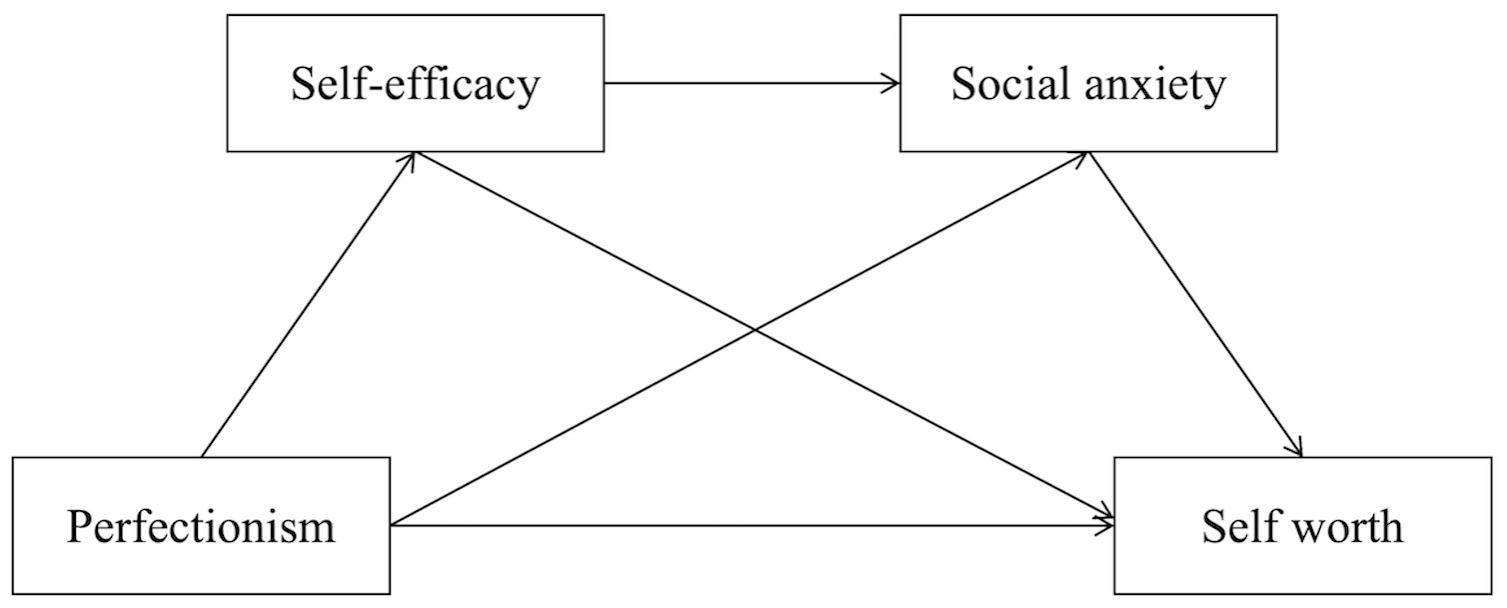
Conceptual serial mediation model linking perfectionism, self-efficacy, social anxiety, and self-worth.

Based on the conceptual framework shown in Figure 1, the study tested the following hypotheses:

The model specifies negative associations of perfectionism with self-worth and self-efficacy, a positive association of perfectionism with social anxiety, a positive association of self-efficacy with self-worth, a negative association of self-efficacy with social anxiety, and a negative association of social anxiety with self-worth (Luo et al., 2021). It further tests whether self-efficacy and social anxiety account for independent and serial indirect associations between perfectionism and self-worth. Because the analysis uses a global perfectionism score, conclusions concern overall perfectionistic tendencies and should not be generalized to perfectionistic strivings and concerns separately.

## 3 Methods

### 3.1 Participants and procedure

This cross-sectional questionnaire study examined associations among perfectionism, self-efficacy, social anxiety, and self-worth in student basketball referees from Sichuan Province, Chongqing Municipality, Guizhou Province, and Yunnan Province. Sampling was stratified by province; 2–3 universities were randomly selected within each province, for a total of 10 institutions, and students were sampled across grades and departments within each institution. The sampling frame was limited to participating universities in Southwest China and should not be interpreted as nationally representative. Participants were 18–22 years old (*M* = 20.01, SD = 1.12): 75 were aged 18, 213 were aged 19, 288 were aged 20, 193 were aged 21, and 91 were aged 22; 568 participants were male and 292 were female.

Inclusion criteria were: (1) age between 18 and 22 years; (2) at least 1 year of basketball refereeing experience (including intra-school, district/county, or higher-level competitions); (3) currently engaged in basketball referee learning, training, or practice; (4) voluntary participation and signed informed consent.

Exclusion criteria were: (1) previous diagnosis of a serious mental illness or current psychological treatment; (2) questionnaire completion time less than 3 min (based on a pretest of 30 participants with a median completion time of 5 min and 20 s, 3 min was set as the minimum valid threshold); (3) obvious patterned responses; (4) failure to correctly answer an embedded attention check item.

The questionnaire was distributed online through the WJX platform, and the first page provided information about the purpose of the human-participant study, anonymity, confidentiality, voluntary participation, and the right to withdraw. Participants entered the study only after selecting “agree and start answering,” and the survey explicitly stated that responses would not affect referee grading, course grades, or ability evaluations. The Ethics Committee of Chongqing University of Education approved the study (Approval No. CQUE202512260020), and the procedures followed the Declaration of Helsinki. Of 940 distributed questionnaires, 860 met the stated quality criteria, yielding a valid response rate of 91.4%; institution-specific response rates were not retained, which limits assessment of institutional nonresponse bias.

### 3.2 Measures

#### 3.2.1 Perfectionism

Perfectionism was assessed with 27 items from the Frost Multidimensional Perfectionism Scale, covering concern over mistakes, organization, parental expectations, personal standards, and doubts about actions (Stöber, 1998). Sample items were “If I fail at work/study, I feel that I am a total failure as a person,”, “I have extremely high goals,” and “Even when I do things carefully, I often feel that I have not done them quite correctly.” Item responses were summed to produce the global score used in the reported analyses; higher totals indicate stronger overall perfectionistic tendencies. The total-score approach does not distinguish perfectionistic strivings from perfectionistic concerns and is therefore interpreted as a limitation rather than evidence that all dimensions function identically. Cronbach's alpha was 0.915 in the present sample.

#### 3.2.2 Self-worth

Self-worth was measured with the six-item self-worth subscale of the Chinese Adolescent Positive Development Scale using a five-point response format from 1 “completely disagree” to 5 “completely agree” (Tong-Ceng et al., 2014). Sample items were “I hold a positive attitude toward myself” and “I feel that I am a person of worth, at least on an equal plane with others”. Items were summed to a total score ranging from 6 to 30, with higher scores indicating greater self-worth; Cronbach's alpha was 0.878.

#### 3.2.3 Self-efficacy

Self-efficacy was measured with 13 items across creative, persuasion, and adaptive self-efficacy, each rated on a seven-point scale. Sample items were “I am confident in my ability to solve problems creatively,” “I feel confident when defending a point of view at work,” and “No matter what changes occur in the organization, I believe I can cope” (Ng and Lucianetti, 2016). The 13 items were summed to a total score ranging from 13 to 91, with higher scores indicating greater comprehensive self-efficacy, Cronbach's alpha was 0.856. The total score was retained because it was the score used in the original analysis and should be interpreted as broad rather than referee-specific self-efficacy.

#### 3.2.4 Social anxiety

Social anxiety was measured with six items rated from 1 (extremely uncharacteristic) to 5 (very characteristic; Alkis et al., 2017). Sample items were “In new situations, I need quite some time to overcome shyness,” “When everyone is watching me, I tend to mess things up,” and “I often feel anxious when speaking in front of a crowd.” Items were summed to a total score ranging from 6 to 30, with higher scores indicating greater social anxiety; Cronbach's alpha was 0.898.

Across the four measures, Cronbach's alpha values ranged from 0.856 to 0.915, indicating good internal consistency in the present sample. Internal consistency alone does not establish construct or discriminant validity, and the available files did not include item-level confirmatory factor analysis, composite reliability, average variance extracted, HTMT, or Fornell-Larcker statistics. Accordingly, the revision does not claim that construct validity has been fully established, and these analyses should be completed with the item-level dataset before final journal resubmission.

### 3.3 Common method bias test

Because all constructs were measured with self-report questionnaires, procedural steps were used to reduce common method variance, including anonymity, non-evaluative instructions, and the statement that there were no right or wrong answers (Tehseen et al., 2017). (Revision—R1-M2: clarified procedural controls). Harman's unrotated single-factor analysis identified 10 factors with eigenvalues greater than 1, and the first factor explained 22.48% of the variance, after rotation, the 10 factors explained 64.5% cumulatively. As an auxiliary construct-level diagnostic calculated from the reported correlation matrix, full-collinearity VIF values ranged from 1.23 to 1.53. Neither Harman's test nor construct-level VIFs can rule out common method variance, so these results are treated as preliminary diagnostics rather than proof that same-source bias is absent. Multi-wave, multi-source, marker-variable, or common-latent-factor approaches are recommended in future research.

### 3.4 Data analysis

Analyses were conducted in SPSS 26.0 with the PROCESS macro. Descriptive statistics, Cronbach's alpha, Pearson correlations, and bivariate linear regression models were first examined. PROCESS Model 6 then tested the parallel and serial indirect associations of self-efficacy and social anxiety between perfectionism and self-worth using 5,000 bootstrap samples (Gao et al., 2026). An indirect association was considered statistically significant when its 95% bootstrap confidence interval excluded zero, and two-tailed statistical significance was set at *p* < 0.05. The primary model did not include demographic, province, certification, or officiating-experience covariates; residual confounding therefore cannot be excluded. Residual normality, linearity, and homoscedasticity diagnostics were not available in the retained summary output and were not reconstructed.

## 4 Results

### 4.1 Descriptive statistics and correlations

Table 1 presents means, standard deviations, and Pearson correlations for the 860 participants. All six nonredundant correlations were statistically significant in the hypothesized directions: perfectionism was negatively correlated with self-worth and self-efficacy and positively correlated with social anxiety; self-efficacy was positively correlated with self-worth and negatively correlated with social anxiety; and social anxiety was negatively correlated with self-worth.

**Table 1:** Descriptive statistics and correlation matrix for each variable.

Variable	*M*	SD	Perfectionism	Self worth	Self-efficacy	Social anxiety
Perfectionism	81.00	21.28	1			
Self worth	18.00	6.69	−0.354^**^	1		
Self-efficacy	52.00	15.77	−0.340^**^	0.428^**^	1	
Social anxiety	18.00	6.90	0.342^**^	−0.523^**^	−0.405^**^	1

N = 860. Values are Pearson correlations; all nonzero coefficients are two-tailed p < 0.001. ^**^p < 0.01.

Mean total scores were 81.00 for perfectionism, 18.00 for self-worth, 52.00 for self-efficacy, and 18.00 for social anxiety. The correlations were moderate in magnitude and support examination of the proposed indirect associations, but they do not establish temporal ordering or causal pathways.

The negative correlation between self-efficacy and social anxiety is consistent with the view that stronger competence beliefs are associated with less threat in evaluative situations. This association provides a statistical basis for evaluating the serial model while remaining compatible with reciprocal or unmeasured explanations.

### 4.2 Regression analysis

Table 2 reports three bivariate regression models in which self-worth was separately regressed on perfectionism, self-efficacy, and social anxiety. The table now provides standardized coefficients, standard errors, 95% confidence intervals, *t* statistics, exact *p* values, VIF values, model *R*^2^, adjusted *R*^2^, and F statistics. These bivariate models describe individual associations with self-worth; the direct and indirect associations in the serial mediation model are reported separately in Table 3.

**Table 2:** Bivariate regression models predicting self-worth.

Variable	Self worth				
	β	T	F	R^2^	R^2^_*adj*_
Perfectionism	−0.354	−11.101^***^	123.227	0.126	0.125^***^
Self-efficacy	0.428	13.863^***^	192.172	0.183	0.182^***^
Social anxiety	−0.523	−17.967^***^	322.826	0.273	0.273^***^

^***^p < 0.001.

**Table 3:** Direct and indirect associations in the serial mediation model.

Path	Effect estimate	SE/Bootstrap SE	95% LLCI	95% ULCI
Total association	−0.2517	0.0238	−0.2984	−0.2050
Direct association	−0.1313	0.0238	−0.1781	−0.0845
Total indirect association	−0.1204	0.0136	−0.1484	−0.0944
Indirect effect 1 (Perfectionism)	−0.0535	0.0098	−0.0733	−0.0354
Indirect effect 2 (Social anxiety)	−0.0375	0.0076	−0.0536	−0.0235
Indirect effect 3 (Self-efficacy → Social anxiety)	−0.0293	0.0052	−0.0401	−0.0197

Indirect association 1: Perfectionism → self-efficacy → self-worth.

Indirect association 2: Perfectionism 81 → social anxiety 81 → self-worth.

Indirect association 3: Perfectionism 81 → self-efficacy 81 → social anxiety 81 → self-worth.

N = 860; 5,000 bootstrap samples. An indirect association is statistically significant when its 95% bootstrap confidence interval excludes zero. The significant direct association indicates partial mediation.

In the bivariate models, higher perfectionism was associated with lower self-worth (β = −0.354), higher self-efficacy was associated with higher self-worth (β = 0.428), and higher social anxiety was associated with lower self-worth (β = −0.523). The narrow confidence intervals and exact *p* values in Table 2 indicate statistical precision in this sample, while the bivariate design does not account for overlap among predictors.

The mediation model in Table 3 addresses the predictors jointly and shows that the direct association between perfectionism and self-worth remained statistically significant after self-efficacy and social anxiety were included. Accordingly, the evidence is consistent with partial mediation rather than a complete disappearance of the direct association.

### 4.3 Chain mediation effect test

**Figure 2:**
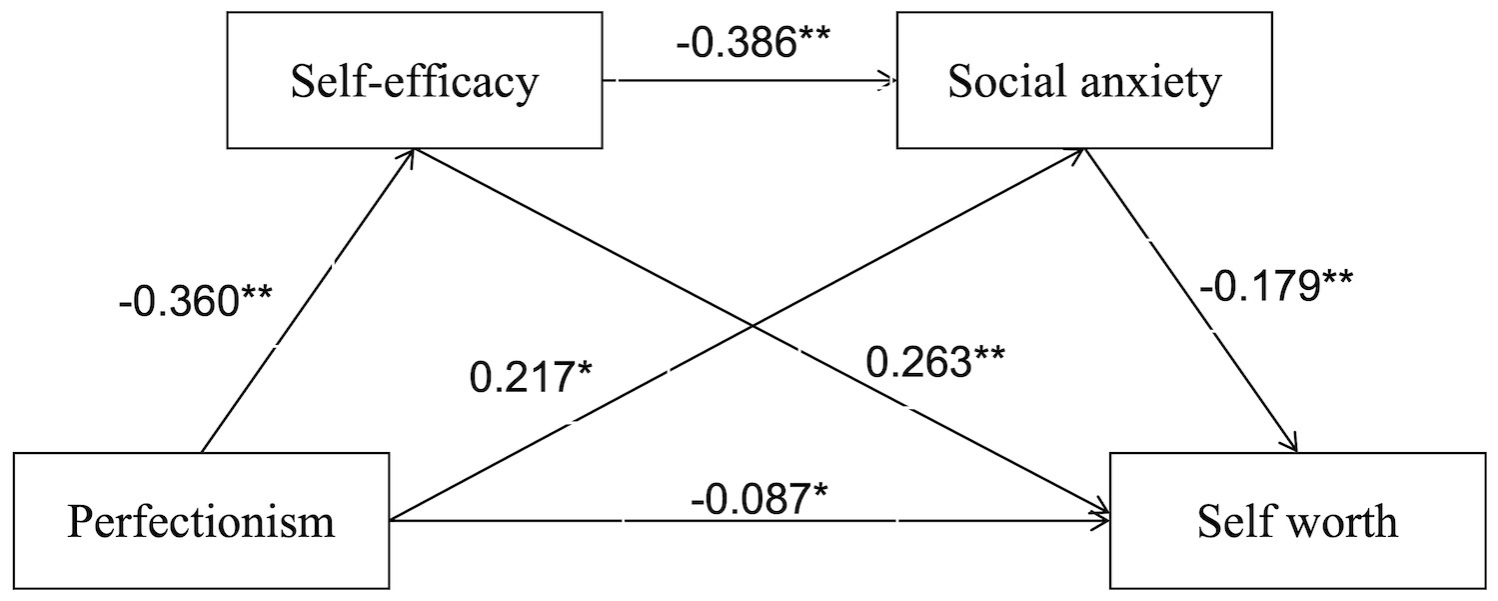
Summary of the reported direct, total indirect, and specific indirect associations.

PROCESS Model 6 was used to estimate the direct, total indirect, specific indirect, and total associations shown in Table 3 and summarized in Figure 2. The total association between perfectionism and self-worth was −0.2517 [95% CI (−0.2984, −0.2050)], and the direct association after inclusion of the mediators was −0.1313 [95% CI (−0.1781, −0.0845)]. Because the direct confidence interval excluded zero, the model indicates partial mediation.

The total indirect association was −0.1204 [95% bootstrap CI (−0.1484, −0.0944)]. The specific indirect associations were −0.0535 through self-efficacy [95% CI (−0.0733, −0.0354)], −0.0375 through social anxiety [95% CI (−0.0536, −0.0235)], and −0.0293 through the serial self-efficacy-to-social-anxiety pathway [95% CI (−0.0401, −0.0197)].

## 5 Discussion

### 5.1 Relationship between perfectionism and self-worth

Higher global perfectionism was moderately associated with lower self-worth among student basketball referees, consistent with research linking perfectionistic concerns to contingent or unstable self-evaluation (Fearn et al., 2022; Wang et al., 2022). The finding should not be interpreted as evidence that high standards are uniformly harmful, because the measure combined dimensions that may have different psychological functions. Rather, the negative association is most plausibly interpreted in relation to concern over mistakes, doubts about actions, and self-critical evaluation, while recognizing that the present total score cannot isolate those components.

Refereeing values precision, fairness, and consistency, so high standards can support preparation and professionalism (Samuel, 2020). Risk may arise when standards become inflexible and every controversial call is treated as evidence of personal inadequacy, a pattern consistent with work on perfectionistic self-presentation and contingent self-worth (Flett and Hewitt, 2014; McArdle and Duda, 2008). Student referees may be especially vulnerable because limited experience gives them fewer stable mastery experiences with which to contextualize errors.

The result suggests that strict technical standards should be embedded in a developmental learning climate. Feedback can identify errors while also distinguishing a correctable decision process from a global judgment about the referee's worth. This framing is compatible with international referee-development research emphasizing structured feedback and progressive expertise rather than immediate error-free performance (Cunningham et al., 2025).

The observed effect was moderate rather than deterministic: many highly perfectionistic students may appear diligent and responsible while privately experiencing rumination or unstable self-evaluation. Coaches and instructors should therefore attend not only to visible effort but also to fear of mistakes, post-game rumination, and dependence on external approval.

### 5.2 Mediating role of self-efficacy

Self-efficacy showed the largest of the three specific indirect associations between perfectionism and self-worth. This pattern is consistent with social cognitive theory: students who discount competent performances and focus on errors may develop weaker mastery beliefs, and weaker mastery beliefs are associated with lower self-worth (Ashrafzade and Sheikhalizadeh, 2023; Bandura, 2001).

Referee training can address this pathway through graded mastery experiences, progressively difficult simulated games, video-assisted review, peer modeling, and feedback that identifies both successful decisions and specific points for improvement. A mentoring program can pair novice referees with experienced officials who model communication, conflict management, and recovery after disputed calls. These strategies operationalize Bandura's sources of efficacy while remaining consistent with the correlational evidence.

Self-efficacy may be a more immediately modifiable training target than a broad personality tendency. Repeated successful practice in rule application, positioning, communication, and conflict management may strengthen competence beliefs even when students retain high personal standards (Stoeber et al., 2008). Experimental evaluation is required to determine whether these activities actually change self-efficacy or self-worth.

Debriefing should also support cognitive restructuring by helping students replace global conclusions such as “I am not good enough” with specific, improvable explanations involving positioning, viewing angle, timing, or communication (Wood et al., 2000). Brief pre-game breathing, attentional routines, and stress-management exercises may further help students apply these revised appraisals under pressure. These proposals are practice implications derived from theory and should not be presented as interventions validated by the present study.

Social anxiety also showed a significant indirect association between perfectionism and self-worth. The result is consistent with international evidence that perfectionism is associated with fear of evaluation and that sport officials operate in unusually public and socially reactive environments (Vargas et al., 2025; Wang et al., 2022). For inexperienced referees, disagreement from players, coaches, spectators, peers, or senior officials may be interpreted as a judgment of personal competence rather than as task-specific information.

Social anxiety may be associated with lower self-worth through selective attention to criticism and reduced willingness to engage in demanding assignments or post-game discussion (Wolfson and Neave, 2007). Such avoidance can reduce access to feedback, mentoring, and successful experiences that might otherwise support competence and belonging. Reducing social anxiety therefore means helping students interpret evaluation more accurately, not encouraging indifference to standards or feedback.

Although the social-anxiety indirect association was smaller than the self-efficacy indirect association, repeated exposure to public evaluation may make its cumulative importance substantial over a referee's development. This possibility should be examined longitudinally because the present data cannot show whether anxiety accumulates or precedes changes in self-worth.

Anxiety may also coincide with narrower attention, less decisive signals, and increased sensitivity to further questioning, creating a plausible feedback loop between emotional state and officiating performance (Carver and Scheier, 1988). Because objective performance was not measured, this proposed loop remains a hypothesis for studies combining psychological measures with video ratings, supervisor evaluations, and decision-accuracy indicators.

### 5.4 Interpretation of the chain mediation mechanism

The serial indirect association was statistically significant: higher perfectionism was associated with lower self-efficacy, lower self-efficacy with higher social anxiety, and higher social anxiety with lower self-worth. The model integrates social cognitive theory with evaluation-based accounts of social anxiety by placing competence beliefs between perfectionistic evaluation and social threat. This interpretation extends referee research by showing how a personality-related tendency, a task belief, and an evaluative emotion may be linked within one statistical model.

A useful conceptual sequence is standard pressure → competence appraisal → evaluative threat → self-worth. Perfectionistic concern may keep attention on gaps and errors; weaker competence appraisal may make questioning feel less manageable; and greater evaluative threat may be associated with lower self-worth (Buckingham et al., 2012; Chasson, 2024). The public, interactive nature of refereeing makes this serial interpretation more contextually specific than a generic parallel-mediation account.

The direct association between perfectionism and self-worth remained significant after both mediators were included. The results therefore support partial mediation: self-efficacy and social anxiety account for a substantial indirect component, but they do not fully explain the association. Other processes, including self-criticism, coping style, perceived support, and unmeasured contextual factors, may contribute to the remaining direct association.

Self-efficacy and social anxiety are related but also retain distinct indirect associations. Lower self-efficacy may make public evaluation feel more threatening, while social anxiety may affect self-worth through attention, avoidance, and perceived social acceptance (Wan et al., 2024). The significant direct association means that training focused on competence and anxiety may be useful but should not be assumed to eliminate all perfectionism-related differences in self-worth.

### 5.5 Limitations and future directions

Several limitations constrain interpretation. First, the cross-sectional design cannot establish temporal order or causal direction; the mediation estimates are statistical decompositions of contemporaneous associations, and reciprocal pathways are possible. Longitudinal, cross-lagged, or experimental designs are required to test whether perfectionism precedes changes in self-efficacy, social anxiety, or self-worth (Collins, 2006). Second, exclusive reliance on self-report increases the possibility of social-desirability and common method variance, and the available Harman and VIF diagnostics cannot rule out this bias.

Third, the measurement analysis reported Cronbach's alpha but did not include CFA, CR, AVE, HTMT, or Fornell-Larcker tests, construct validity and discriminant validity therefore require further verification with item-level data. Fourth, the sample was drawn from 10 participating institutions in Southwest China, and institution-specific response rates were unavailable; selection, regional, and cultural specificity limit national and international generalizability. Fifth, the primary model did not control for gender, academic year, province, certification level, officiating experience, or number of competitions, so residual confounding and subgroup differences remain possible.

Sixth, the study did not include behavioral or objective indicators of referee performance. Future studies should combine questionnaires with game-video ratings, supervisor evaluations, decision accuracy, communication quality, and referee-grade progression. Such data would test whether the psychological associations observed here are also related to officiating performance and sustained participation.

Seventh, the reported analysis treated perfectionism as a global score even though perfectionistic strivings and perfectionistic concerns may have different or opposing associations. Future item-level analyses should examine these dimensions separately and test whether concern over mistakes and doubts about actions are more strongly associated with lower self-worth than personal standards or organization. This distinction would help training programs avoid treating all high standards as maladaptive.

Finally, environmental factors such as supervisor support, mentoring quality, team climate, and competition culture were not measured. Multilevel and longitudinal studies could test whether supportive environments weaken the associations of perfectionistic concerns with anxiety and lower self-worth. These additions would provide a more complete account of referee development than an individual-level cross-sectional model alone.

## 6 Conclusions

Among 860 college student basketball referees, higher global perfectionism was associated with lower self-worth, lower self-efficacy, and higher social anxiety. Self-efficacy and social anxiety showed significant parallel and serial indirect associations, while the direct association between perfectionism and self-worth remained significant; the results therefore indicate partial mediation. The findings support further evaluation of graded mastery practice, constructive debriefing, cognitive restructuring, stress management, and mentoring as training components, but the present cross-sectional study does not establish their effectiveness. Longitudinal, experimental, multidimensional, and multi-source research is needed to verify temporal ordering, construct validity, subgroup differences, and links with objective officiating performance.

## Data Availability

The original contributions presented in the study are included in the article/Supplementary material, further inquiries can be directed to the corresponding author.
